# Modality Matters: Intact and Enhanced Memory Skills in Children From High‐Stress Environments

**DOI:** 10.1111/cogs.70214

**Published:** 2026-04-29

**Authors:** Gabriele Paone, Arran J. Davis, Emma Cohen

**Affiliations:** ^1^ Centre for the Study of Social Cohesion School of Anthropology and Museum Ethnography University of Oxford; ^2^ St. John's College University of Oxford; ^3^ Wadham College University of Oxford

**Keywords:** Evolutionary anthropology, Adaptation, Children, Memory, Hidden talents model, Harsh and unpredictable environments

## Abstract

Although adverse ontogenetic environments are associated with potential impairments in children's memory, recent research suggests that individuals can develop specialized skills to navigate such settings. We conducted a study on short‐term memory (STM) and working memory (WM) among 357 children (176 females, *M_age_* = 8.23 years, *SD_age_* = 1.49 years) from two environments in Naples (Italy): Scampia, a neighborhood characterized by chronic socioeconomic hardship, and Pozzuoli, a comparatively lower‐stress area. In Part 1, which used conventional abstract stimuli, Scampia children performed similarly to the Pozzuoli control group. In Part 2, which used social stimuli, Scampia children outperformed Pozzuoli peers in both STM and WM. These findings highlight the complexity of memory development, showing that children from high‐stress environments can exhibit intact or even enhanced skills that are functionally relevant to the challenges of their surroundings.

## 1 Introduction

Millions of children worldwide face adversities such as social insecurity, poverty, conflict, or displacement (WHO, 2024). These conditions are widely recognized as developmental risk factors, and are often linked to impairments in cognition, behavior, and mental health (e.g., Lown, Korcha, & Greenfield, 2011; Brito, Fifer, Myers, Elliott, & Noble, 2016; Kim et al., 2013; Lorant et al., 2003; Perez & Widom, 1994; Schwabe & Wolf, 2010; Shin, Hong, & Hazen, 2010; Strine et al., 2012). Although there is robust evidence on the potential developmental risks posed by adversity, this is only part of the picture. Among many of these children, there is also evidence of *resilience* and development considered typical by Western standards (see Benard, 1995; Goldstein & Brooks, 2013; Masten, 2001).

More recently, approaches informed by evolutionary‐developmental theory have shifted the focus from deficit and resilience to adaptation. These perspectives propose that humans respond adaptively to adversity through strategies that increase their survival, for example, through group cohesion and cooperation (see Ember, Skoggard, Ringen, & Farrer, 2018; Gelfand, Nishii, & Raver, 2006; Whitehouse et al., 2017). A novel and more fine‐grained framework, the *hidden talents model* (HTM; see Ellis et al., 2022), suggests that high‐stress environments may also foster particular cognitive and behavioral skills that are adaptive within these contexts. The model aligns with broader theories of developmental plasticity, which describe how organisms adjust neurocognitive development in response to environmental input (Bjorklund & Ellis, 2014; Ellis & Del Giudice, 2014; Frankenhuis & Amir, 2022; Frankenhuis & de Weerth, 2013).

This reframing has opened new avenues for research into context‐specific development. For example, traits like impulsivity or risk‐taking, often deemed maladaptive in formal education settings, may serve important problem‐solving functions in unpredictable environments. Unlike resilience models, which emphasize typical development in spite of adversity, the HTM focuses on context‐specific cognitive and behavioral skills that emerge as adaptive responses, and often remain “hidden” unless formally recognized or assessed (Ellis, Bianchi, Griskevicius, & Frankenhuis, 2017, 2023).

Despite its conceptual potential, empirical research on stress‐adapted skills remains limited due to significant methodological and ethical challenges (Ellis et al., 2017; Frankenhuis, Young, & Ellis, 2020). Experimental manipulation of early life stress is not ethically viable in human populations, and much of the literature relies on other animal models (e.g., Calandreau et al., 2011; Chaby et al., 2015; Farine, Spencer, & Boogert, 2015). Even where laboratory studies simulate stress, the conditions often diverge substantially from real‐world adversity, limiting external validity (e.g., Dickerson & Kemeny, 2004). Naturalistic research in high‐stress environments offers an alternative path, but comes with its own constraints related to research control, logistics, and risk to researchers.

### 1.1 Short‐term and working memory

Among the cognitive skills potentially shaped by early adversity (for a review, see Ellis et al., 2017, 2023), this study focuses on short‐term memory (STM) and working memory (WM) capacity. STM refers to the temporary storage and retrieval of information, serving as a repository for data that needs to be promptly accessed. WM involves not only storage and retrieval, but also the active manipulation of momentary information for more demanding cognitive tasks (Alloway, Gathercole, Willis, & Adams, 2004; Baddeley, 2012; Baddeley, Eysenck, & Anderson, 2020; Baddeley & Hitch, 1974; Gathercole, Pickering, Knight, & Stegmann, 2004). This operationalization is commonly employed in research and aligns with the task structure utilized in the present study (although the distinction continues to be a topic of debate, see Aben, Stapert, & Blokland, 2012).

We targeted STM and WM for four reasons. First, they play a central role in children's cognitive development and contribute to learning, comprehension, problem‐solving, and general intelligence (Cowan, 2014). Evidence of adaptive developmental differences in these systems could, therefore, inspire further research and applications in child development.

Second, like other executive functions, STM and WM are closely linked to the prefrontal cortex (Diamond, 2013; Friedman & Robbins, 2022), which undergoes protracted development from childhood through early adulthood (e.g., Giedd et al., 1999; Tanaka, Matsui, Uematsu, Noguchi, & Miyawaki, 2012). Given this extended neural maturation, STM and WM are likely significantly shaped by environmental influences, including stress exposure (Farah et al., 2006). Consistent with this view, numerous studies have documented a negative relationship between exposure to acute and/or prolonged stress and performance on conventional STM and WM tasks (e.g., Brand, Hanson, & Godaert, 2000; Bremner et al., 1993; Evans & Schamberg, 2009; Farah et al., 2006; Hart & Rubia, 2012; Klein & Boals, 2001; Tine, 2014).

Third, recent research has provided a more nuanced insight, suggesting that different components of STM and WM may show different adaptive profiles under adversity, such as enhancements in specific WM aspects like rapid tracking and memory updating, but impairments in others, such as WM in the presence of distractions (Young, Griskevicius, Simpson, Waters, & Mittal, 2018), or superior STM and WM in stress‐exposed individuals under specific conditions (Nweze, Nwoke, Nwufo, Aniekwu, & Lange, 2020). This emerging literature indicates that adversity does not uniformly impair memory; rather, it may selectively strengthen some processes while weakening others, depending on contextual factors. Importantly, evidence for stress‐adapted STM and WM skills has so far focused primarily on updating rather than on capacity (Ellis et al., 2023). Capacity‐based measures—typically assessed using abstract span tasks—have often been interpreted as reflecting stress‐related deficits, whereas updating processes have been proposed as loci of potential stress‐adapted strengths (e.g., Young et al., 2018). As a result, it remains unclear whether capacity itself is uniformly impaired under adversity, or whether its apparent vulnerability depends on the nature and ecological relevance of the material being processed.

Fourth, trade‐offs have been reported *between* cognitive functions more broadly: for example, children exposed to unstable caregiving show reduced response inhibition and attentional control but enhanced cognitive flexibility (Fields et al., 2021), and adults with more unpredictable childhoods perform worse on inhibition but better on shifting, particularly under experimentally induced uncertainty (Mittal, Griskevicius, Simpson, Sung, & Young, 2015). Although these latter studies do not focus on memory, they illustrate that adversity‐related cognitive profiles need not be globally impaired; instead, particular components may be strengthened, while others are diminished.

These studies have raised new questions about the variability and complexity of STM and WM systems within individuals and across diverse developmental contexts. Our research builds on this literature by asking whether a similar pattern appears in STM and WM capacity itself: adversity‐exposed children may show reduced capacity for conventional abstract material, but preserved or enhanced capacity for ecologically salient social information (e.g., faces and interpersonal cues). In this sense, we test whether task modality and ecological relevance reveal context‐specific strengths within a system often framed in purely deficit terms.

### 1.2 The present study

Building on these theoretical and empirical foundations, we conducted a natural experiment in two parts with primary school‐aged children across two sites in Naples (Italy)—a high‐stress environment (Scampia) and a lower‐stress control environment (Pozzuoli, see descriptions below).

Part 1 employed conventional memory span tasks with abstract verbal and visuo‐spatial stimuli (i.e., digit strings, geometric figures) to assess STM and WM. Based on prior research, we hypothesized that children from Scampia would score lower than those from Pozzuoli on these tasks.

In Part 2, we adapted this span paradigm to include socially meaningful stimuli—specifically, unfamiliar faces—to assess STM and WM for social information. This decision was driven by three considerations. First, most cognitive and developmental research is based on Western, educated, industrialized, rich, and democratic samples (W.E.I.R.D., Henrich, Heine, & Norenzayan, 2010), which tend to experience relatively stable and predictable environments, socioeconomic security, and widespread access to formal education. As a result, commonly studied variables and assessment tools may fail to capture skills developed outside these contexts. Second, neuroscientific research suggests the existence of STM and WM systems dedicated to processing social information (e.g., Meyer, Spunt, Berkman, Taylor, & Lieberman, 2012), which are typically overlooked in conventional STM and WM assessment with abstract stimuli. Third, a large body of research shows that organisms more readily learn associations that are fitness‐relevant, especially those regarding threat (Öhman & Mineka, 2001; Seligman, 1971). For instance, nonhuman animal studies suggest that low social rank and/or environmentally harsh rearing conditions predict improved memory for threatening stimuli (Bagot et al., 2009), and in humans, children from culturally distinct populations rapidly learned and remembered which animals are dangerous, whereas other concurrently presented information (such as names and diets) was largely forgotten after a single exposure (Barrett & Broesch, 2012).

In line with this, the hidden talents model proposes that developmental systems may calibrate cognitive abilities toward the kinds of problems that must be solved under adversity, including monitoring social threats and opportunities (Ellis et al., 2023; Frankenhuis, de Vries, Bianchi, & Ellis, 2020). In low socioeconomic status (SES) contexts, where people have limited control over structural conditions (e.g., poverty, food insecurity), life outcomes often depend heavily on external social forces and on other people (Kraus, Piff, Mendoza‐Denton, Rheinschmidt, & Keltner, 2012). Developmental adaptations may, therefore, bias attention, learning, and memory toward these individuals and the social information they convey.

Consistent with this view, several studies report selective advantages in social‐information processing among individuals exposed to adversity. These include better memory and reasoning about social dominance in dangerous neighborhoods (Frankenhuis et al., 2020), enhanced memory for affectively salient or threatening stimuli under caregiving risk (Rifkin‐Graboi et al., 2018), greater incidental memory for faces among low‐SES participants (Dietze, Olderbak, Hildebrandt, Kaltwasser, & Knowles, 2022), more accuracy in discerning trustworthiness among peers in post‐institutionalized youth (Pitula, Wenner, Gunnar, & Thomas, 2017), or higher empathic accuracy among lower SES adults (Kraus, Côté, & Keltner, 2010). Such abilities may provide an adaptive advantage in harsh and unpredictable environments, where the costs of incorrectly interpreting or remembering social information—such as others’ dominance—are likely to be higher than in safer environments (Frankenhuis et al., 2020). Despite these advances, most empirical work on attunement to social information under adversity has focused on adults rather than children (Ellis et al., 2023). Much less is known about when such specialized skills emerge, or how they manifest in childhood.

Taken together, this theoretical and empirical work suggests that STM and WM for social information may be particularly sensitive to environmental demands, making them strong candidates for context‐specific strengths. In a context like Scampia, where monitoring others’ identities, alliances, and potential threats is part of everyday life (see description below), we hypothesized that children would outperform their Pozzuoli peers on social memory tasks. Age and sex were included as covariates to examine their influence on performance, though they were not central to our hypotheses.

To complement cognitive data, we also administered the Perceived Stress Scale for Children (PSS‐C, White, 2014) to measure participants’ subjective experience of stress, and conducted ethnographic fieldwork to contextualize developmental conditions across the two sites.

## 2 Research sites

This study was conducted across two distinct environments in the Naples metropolitan area: Scampia neighborhood and the comparatively lower‐stress area of Pozzuoli. To assess structural adversity, we refer to the Italian National Institute of Statistics (Istat) Index of Social and Material Vulnerability (IVSM) (Istat, 2020), which integrates indicators such as education levels and economic hardship (see Supplementary Material 1). According to the latest data, Scampia exhibits significantly higher vulnerability (121.4) than Pozzuoli (104.1). The overall score for the city of Naples is 104.9. While the IVSM provides valuable insight into municipal‐level disparities, it can mask important variation within and between urban zones, and does not fully capture the specific forms of adversity experienced by the populations in this study. For instance, Scampia's informal labor economy excludes many residents from formal employment statistics (Orientale Caputo, 2012).

To complement the IVSM and gain a better understanding of these environments, the first author (a native Italian) conducted 9 months of ethnographic fieldwork across the two sites, including schools, community centers, religious events, advocacy meetings, and volunteer work with NGOs supporting the Scampia community. Additionally, over 100 semi‐structured interviews were conducted with parents, teachers, educators, social workers, healthcare professionals, law enforcement officers, clergy members, local workers, former and current gang members, and politicians. This provided firsthand insight into the social and structural challenges shaping children's development, and informs both the contextual descriptions that follow and the interpretation of the results.

**Fig. 1:**
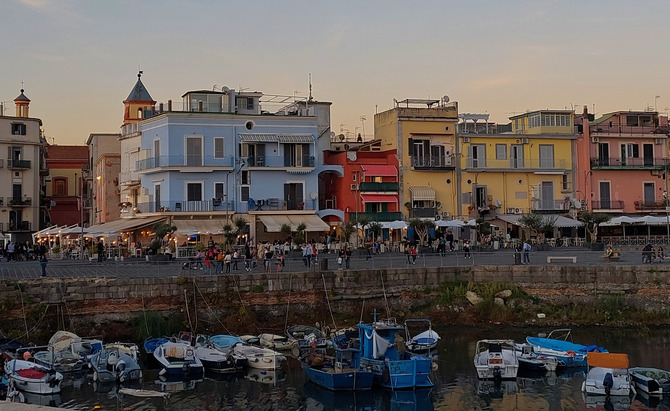
The Pozzuoli dock, with its distinctive promenade and restaurants overlooking the old harbor.

**Pozzuoli**. A coastal city in the metropolitan area of Naples, Pozzuoli, serves as the lower‐stress comparison site in this study (Fig. 1). With approximately 76,000 inhabitants, it is typically middle‐class and a popular tourist destination, renowned for its Roman remains and extensive coastline. Pozzuoli benefits from well‐developed infrastructure and public services, including healthcare, education, policing, sanitation, public transportation, and entertainment. The local administration has received multiple awards for improving urban living standards (see Cronaca Flegrea, 2024; Olcesi, 2017).

Fieldwork revealed a relatively stable and resource‐rich setting for children, with structured daily routines, well‐equipped schools, and access to multiple extracurricular activities. Teachers described the academic setting as largely supportive, despite occasional individual challenges. Pozzuoli residents expressed relatively high trust in local institutions and services, distinct from the pervasive mistrust reported in Scampia. Nearly half of the adult population has completed secondary or higher education (Istat, 2011), and economic stability is reflected in secure employment opportunities for many parents. Although some families face financial difficulties—especially due to rising living costs in Italy (Colombarolli, 2024; European Commission. Directorate‐General for Economic and Financial Affairs, 2024)—concerns expressed by parents centered on future academic or employment prospects, not basic safety or access to primary services, topics far more prominent in discussions with parents from Scampia.

While Pozzuoli offers a relatively secure and structured environment, certain families still face economic difficulties, and some interviewees reported challenges related to youth delinquency and unemployment. Nevertheless, Pozzuoli's stronger structural support, educational opportunities, and general sense of security clearly distinguish it as a lower‐stress environment compared to the other site in this study.

**Fig. 2:**
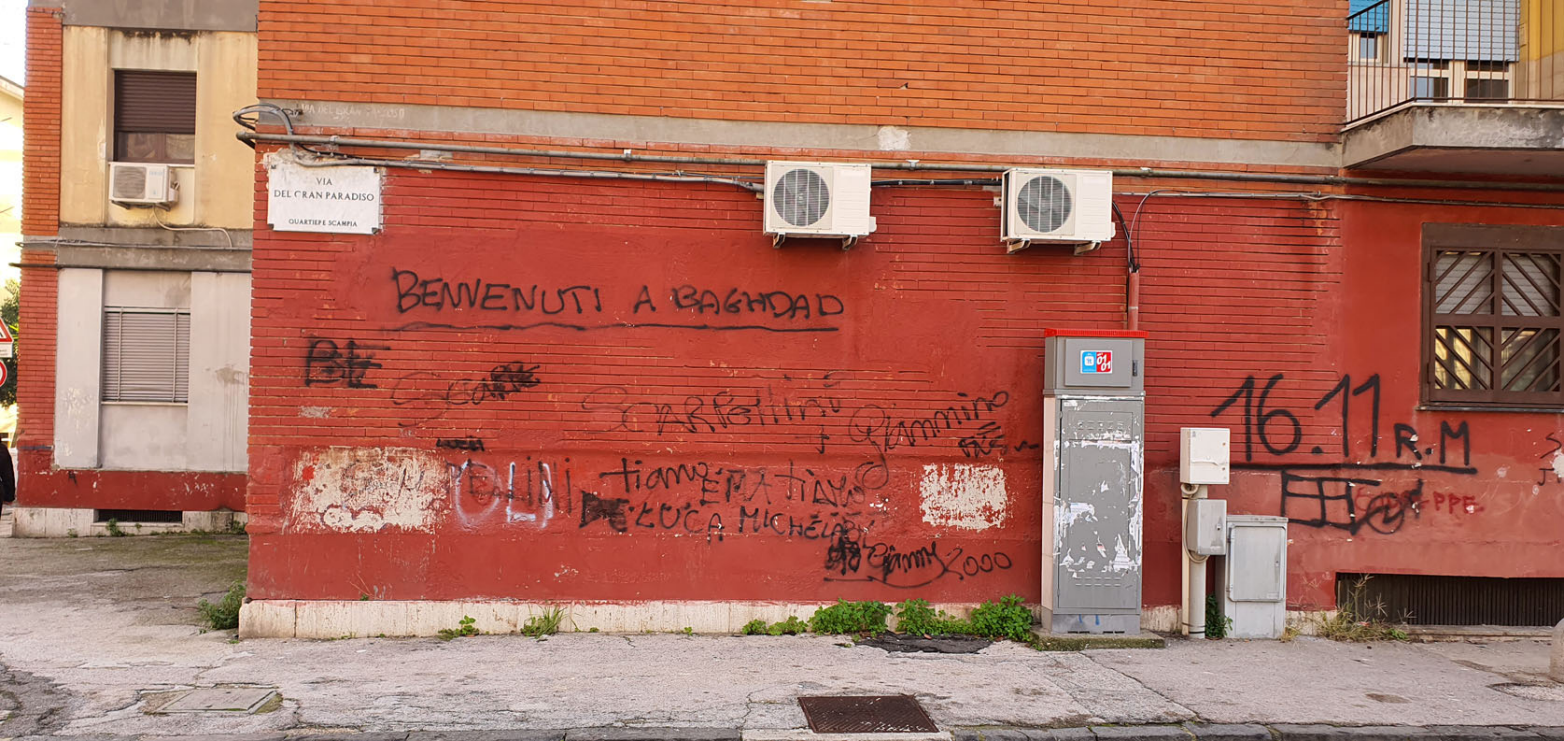
Wall along Via del Gran Paradiso, Scampia. The inscription on the wall reads: “Welcome to Baghdad”, a reference likely intended to evoke war‐zone imagery.

**Scampia**. Situated in the northern periphery of Naples, Scampia is the most notorious among the city's high‐deprivation neighborhoods (Fig. 2). Officially home to around 40,000 people (though many more live in informal housing), it is marked by systemic neglect, high unemployment, and a long‐standing presence of organized crime. Known for its incomplete public housing—particularly the “Vele” complex—Scampia became a stronghold of the Camorra criminal organization in the 1980s and evolved into one of Europe's largest open‐air drug markets (Bertone & Leiduan, 2024; Di Costanzo, 2013; Saviano, 2006).

Violent clan conflicts—particularly the 2004–2005 feud between the Di Lauro clan and the *Scissionisti* splinter group, and a second feud in the 2010s (Di Costanzo, 2013; Saviano, 2006)—fractured territorial control across multiple rival groups, a fragmentation that persists today and remains associated with episodes of violence. Residents describe a social environment in which clan affiliations still shape daily life and require ongoing monitoring of local power dynamics, an aspect that potentially sharpens children's memory for socially relevant information.

Scampia's notoriety drew international media attention, inspiring books, films, and television series (e.g., Saviano's, 2006 best‐seller *Gomorrah* and the homonymous Netflix series). Media exposure did little to reverse Scampia's structural decline, but solidified its image as a center of crime and deprivation—a stigma that persists to this day (Marelli, 2021).

The area continues to suffer from infrastructural decay, physical isolation, and limited access to essential services. Unemployment stands at 46% (compared to a city‐wide 21%), and only 21% of adults have a high school diploma (Istat, 2011). At the time of the research, essential services remain scarce, with few shops and supermarkets, and no public cultural and recreational spaces, except for those run by modest voluntary associations. Many families rely on informal employment and face intergenerational poverty. Aligned with observations in comparable contexts, residents tend to prioritize immediate gains over long‐term investments in education or other ventures (see Griskevicius, Tybur, Delton, & Robertson, 2011), an inclination that aligns with the lure of criminal activities, which offer swift earnings.

Compounding these challenges, Scampia lies within the “Land of Fires,” a region associated with illegal toxic waste dumping and environmental contamination (Di Gennaro & Fagnano, 2015; Legambiente, 2003; Saviano, 2006). This has contributed to severe health issues for residents, including respiratory diseases, cancers, and congenital disorders (Beccaloni et al., 2020; Buonaugurio, 2019; Greco, 2016; Rocco et al., 2016; Senior & Mazza, 2004). Notably, Scampia itself contains an illegal landfill that is frequently alight, intentionally or not, releasing toxic fumes.

Children growing up in Scampia thus face multiple overlapping stressors: criminal activities, economic marginalization, toxic exposure, and institutional neglect. While these are typically associated with impairments in executive functions like STM and WM (Aurino, Wolf, & Tsinigo, 2020; Bogliacino, Grimalda, Ortoleva, & Ring, 2017; Brooks‐Gunn & Duncan, 1997; Calderón‐Garcidueñas et al., 2015; Evans & Schamberg, 2009; Farah et al., 2006; Hackman et al., 2014; Ioar et al., 2024; Lopuszanska & Samardakiewicz, 2020; Mooney, Prady, Barker, Pickett, & Waterman, 2021), no study has systematically examined cognitive outcomes in this context.

## 3 Experimental study

The conditions in Scampia create a highly uncertain and uniquely challenging developmental environment that potentially significantly shape children's cognitive development. Multiple studies link the kinds of stressors they routinely face (e.g., low SES) to various developmental impairments, including STM and WM deficits (e.g., Evans & Schamberg, 2009; Klein & Boals, 2001; Tine, 2014). To empirically investigate STM and WM development in children from Scampia and compare them to a relatively lower‐stress control group (Pozzuoli), we conducted a study in two complementary parts. Part 1 used conventional abstract stimuli to measure STM and WM in children aged 6−10 across the two study sites, while Part 2 focused on social stimuli to assess these memory systems in the same groups. Additionally, we used the PSS‐C (White, 2014) to evaluate the children's self‐reported stress levels and provide a quantitative measure of stress exposure in these environments. We hypothesized that children from Scampia would perform worse on conventional memory tasks (Part 1), but better on social memory tasks (Part 2). These hypotheses and study designs were preregistered on AsPredicted (Part 1: https://aspredicted.org/dj24‐jp5s.pdf; Part 2: https://aspredicted.org/sk2m‐pg5q.pdf). Below, we report Part 1 and Part 2, followed by an exploratory analysis comparing effect sizes across the study samples and memory task modalities (“abstract” and “social”).

### 3.1 Part 1—Conventional assessment of STM and WM (“abstract” memory)

#### 3.1.1 Method

##### 3.1.1.1. Participants

Data were collected from 357 children across the two populations (176 females, *M_age_* = 8.23 years, *SD_age_* = 1.49 years), spanning the first to last grades of Italian primary school, which typically includes children aged 6−10 years. The sample includes 167 children from Scampia (89 females, *M_age_* = 8.25 years, *SD_age_* = 1.50 years), and 190 from Pozzuoli (87 females, *M_age_* = 8.22 years, *SD_age_* = 1.48 years). Sample sizes were determined via power analysis, which indicated that 125 participants from both Scampia and Pozzuoli would be needed to have a power of at least 0.8 to detect differences in memory test span scores of 0.3 points or greater between the two environments (see Supplementary Material 2).

To avoid excluding classmates from participation, all children in the class were allowed to participate; children younger than 5 years old (*n* = 16), older than 11 years (*n* = 1), and those who did not understand the tasks (*n*_verbal STM_ = 3, *n*_verbal WM_ = 17, *n*_visuo‐spatial STM_ = 3, *n*_visuo‐spatial WM_ = 5) were excluded from the analyses. Furthermore, data from participants who failed a prior comprehension check assessing their ability to follow task instructions were excluded from analyses (see “Measures” for details on the task, and Table S1 for details on exclusion by test). Participants completed four tasks (see below for details). The refined sample sizes after exclusions are presented in Table 1.

**Table 1:** Demographic information of participants across tasks

Environment	Task type	*N*	*n_female_* (%)	*M_age_* (years)	*SD_age_* (years)
Scampia	Verbal STM	157	83 (52.9)	8.35	1.41
	Verbal WM	154	81 (52.6)	8.38	1.41
	Visuo‐spatial STM	156	83 (53.2)	8.35	1.42
	Visuo‐spatial WM	156	83 (53.2)	8.35	1.42
Pozzuoli	Verbal STM	180	83 (46.1)	8.34	1.42
	Verbal WM	169	77 (45.6)	8.41	1.42
	Visuo‐spatial STM	181	84 (46.4)	8.33	1.42
	Visuo‐spatial WM	179	84 (46.9)	8.34	1.43

Children were recruited through the schools they attended in their respective neighborhood/city. Oral consent to participate in the research was obtained from the institutions where the study took place and from the teachers. Written consent was obtained from the parents/guardians of the children. Assent was also obtained from the children before the beginning of the session. As a token of appreciation for their participation, children received stickers and a “Young Researcher” certificate. As preregistered, we also collected data from a sample of Roma children (*n* = 47, 15 females, *M_age_* = 8.63 years, *SD_age_* = 1.50 years) living in harsh conditions nearby Naples (for an account of our ethnographic work with this population, see Paone, 2025). However, due to sample size considerations, we do not report results for this population here, but the data are provided on our Open Science Framework (OSF) project website (https://osf.io/yuzcd/). This research was approved by the relevant Departmental Research Ethics Committee of the University of Oxford.

##### 3.1.1.2. Procedure

The sessions took place individually in a quiet room at the school, during school hours. All sessions were administered by the lead author. Children were briefly removed from class, provided this had the class teacher's approval. The session began with the administration of the PSS‐C (White, 2014), followed by the assessment of a specific memory modality. Task order was counterbalanced across participants, with half beginning with Part 1, and the other half with Part 2 (reported below). Part 1 entailed four tasks evaluating verbal STM, verbal WM, visuo‐spatial STM, and visuo‐spatial WM. The order of the tasks was partially counterbalanced, with half of the participants starting with the verbal tasks and the other half with the visuo‐spatial tasks.

Tasks were administered in Italian by the first author (a native Italian speaker). Demographic information about participants (i.e., age, sex, and place of origin) was collected from the school.

**Stress perception**. Defining stress has proven to be challenging, with no universally accepted definition (Levine, 2005). Similarly, gauging stress levels in children presents several difficulties, leading to the proposal of various tools and methodologies. Moreover, assessing stress in very young children (ages 6–10) in harsh and unpredictable environments raises additional methodological and ethical constraints. Standardized measures of adversity used in the hidden talents literature, such as neighborhood violence scales (e.g., Mittal et al., 2015), or detailed questionnaires about history of child abuse and neglect (e.g., Bernstein, Fink, Handelsman, & Foote, 1994), were not suitable for this context. Local gatekeepers, teachers, and community workers advised that questions directly addressing topics such as violence, incarceration, financial hardship, abuse and neglect, or residential instability would be culturally unacceptable, potentially distressing for children, and could compromise participant wellbeing as well as the safety of the researcher.

Given these constraints, we selected the PSS‐C (White, 2014) as the least intrusive and most ethically appropriate option for this population. Although originally developed as a screening tool for children's current stress and anxiety symptoms rather than as a comprehensive assessment of developmental adversity, the PSS‐C nevertheless provides an index of children's subjective stress experience. The scale, derived from the adult version (Cohen, Kamarck, & Mermelstein, 1983), is designed for use with participants aged 5−18 and is acknowledged as a reliable measure of an individual's stress perception at a given moment, and across various age ranges and languages. It covers domains such as time constraints, school performance, friendships, parent/caregiver relationships, experiences of conflict and anger, overall happiness, sleep patterns, and the sense of being loved, which align with established frameworks used to describe childhood stress perception (e.g., Byrne, Thomas, Burchell, Olive, & Mirabito, 2011; Maldonado et al., 2008). To ensure inclusivity, we adapted references to “your parents” by broadening to “your parents or whoever takes care of you at home,” acknowledging that not all children may be cared for by their parents.

Originally in English, the PSS‐C questions were translated into Italian by a bilingual speaker, with back‐translation into English by another bilingual individual to ensure accuracy.

**Short‐term memory and working memory**. STM is typically assessed through a span task, which requires participants to recall items (e.g., digits, words, or figures) in the same order they were presented, a method utilized at least since Ebbinghaus (1885). The evaluation of WM normally couples recall with a concurrent activity, demanding not just retrieval but also the active manipulation of information.

To assess STM, we employed the PML‐2 (D'Amico & Lipari, 2020), a test battery aligned with the traditional Baddeley and Hitch (1974) model. This battery, developed specifically for Italian children, was designed for easy administration across various contexts, ranging from schools to clinical settings.

To assess STM, we employed two tasks from this battery. For verbal STM, we utilized Task 2 “rievocazione seriale di cifre” (serial recall of digits), in which participants are required to repeat a sequence of numbers between 1 and 9 in the order they were presented. A warm‐up exercise introduced the task, in which two digits were presented orally for the child to repeat in the same order. This was used to assess children's task comprehension. If the child failed to recall the sequence correctly after the experimenter, they were given up to two further attempts. Failure to answer correctly in the third attempt resulted in discontinuation of the task, and these children's data were excluded from analyses.

To evaluate visuo‐spatial STM, we used Task 6 “rievocazione di sequenze visuo‐spaziali” (recall of visuo‐spatial sequences) from the battery. The researcher sat beside the child and tapped a series of circles printed on a laminated paper sheet in a specific sequence, which the child was then required to replicate by tapping the circles in the same order. A comprehension check was performed as described above for the digit task.

For both tasks, stimuli were presented/indicated by the experimenter at a rate of 1 per second, beginning with two‐item lists at the first iteration and extending up to eight‐item lists. Each iteration consisted of two different lists, both administered. If the child correctly recalled at least one list, they progressed to a longer one, incrementing by one item at a time. The task ended when both lists of a given length were recalled incorrectly. Correct recall required reproducing all elements in the same order as presented.

Each task generates two scores: the *span* score, which indicates the length of the longest correctly recalled list (e.g., if the child correctly recalled a five‐item list and failed both six‐item lists, their span score was five), and the *total* score, calculated as the sum of all correctly recalled lists (e.g., if the child recalled two two‐item lists and one three‐item list, their total score is 2 + 2 + 3 = 7). The span score was the primary variable of interest for the main analyses. The total score, reported in the Supplementary Material, enables a more fine‐grained evaluation of variation among children who, with similar span measures, may have recalled more or longer lists.

To assess WM, we employed two tasks, one verbal and one visuo‐spatial, that mirrored the STM tasks, but differed in that they required items recall in reverse order. Forward span primarily measures immediate storage and serial recall, processes typically associated with STM (Baddeley, 2012). In contrast, backward span requires children to hold the sequence in mind, mentally reverse its order, and then produce the reversed sequence, engaging continuous manipulation of information in addition to simple storage and retrieval. Because of this additional executive component, backward span tasks are widely used as standard measures of WM (e.g., Alloway, Gathercole, & Pickering, 2006; Berry, Waterman, Baddeley, Hitch, & Allen, 2018; St Clair‐Thompson, 2010). The administration of the WM tasks followed the same procedure as the STM tasks described above, including the prior comprehension check.

#### 3.1.2 Analyses

We ran four linear regression models, one for each of the dependent variables from the four tasks (i.e., verbal STM, visuo‐spatial STM, verbal WM, visuo‐spatial WM). The main predictor variable was participant environment, and we included participant age and sex as covariates. We used treatment contrasts on the environment variable, with Pozzuoli (lower‐stress population) being the reference category. We also used linear regression models to test whether participants’ PSS‐C scores predicted the dependent variables. We did not exclude outlying observations. Participant data were only excluded due to age or if they were unable to follow or complete the memory task (see Tables S1 and S2).

**Table 2:** Span scores for STM and WM tasks across the two populations

Environment	Verbal STM		Verbal WM		Visuo‐spatial STM		Visuo‐spatial WM	
	*M*	*SD*	*M*	*SD*	*M*	*SD*	*M*	*SD*
Pozzuoli	4.36	0.95	2.88	0.83	4.30	1.05	3.70	1.19
Scampia	4.39	1.02	3.01	0.76	4.29	1.08	3.54	0.97

#### 3.1.3 Results

##### 3.1.3.1 Descriptive statistics

Overall, task comprehension was high (see Table S2). The scores on the memory tasks are reported in Table 2.

An ANOVA (*F* = 2.56, *p* = .111) showed that perceived stress scores did not differ significantly between Scampia (*M* = 11.00, *SD* = 4.80) and Pozzuoli (*M* = 10.21, *SD* = 4.31); see Tables S3 and S4.

##### 3.1.3.2 Effects of environment on memory test scores

**Fig. 3:**
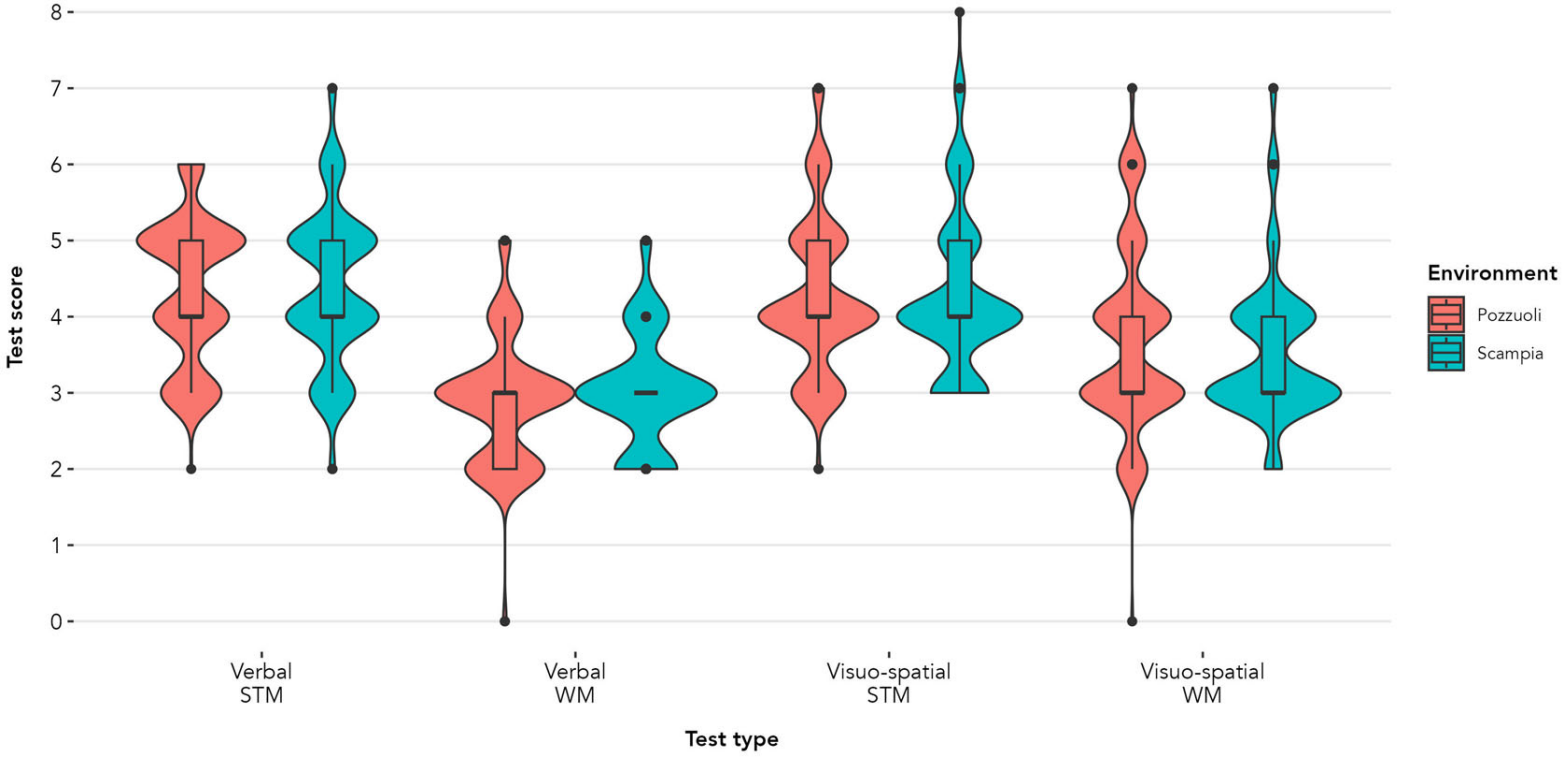
Participants’ span scores by environment and test type. Violin plots show the distribution of scores, with wider sections indicating higher density. Embedded boxplots indicate the median (horizontal line), interquartile range (box), and range (whiskers). Individual dots represent participant scores that fall outside 1.5 times the interquartile range (i.e., outliers).

The scores of Scampia children were not significantly different to the scores of Pozzuoli children across any of the tasks: verbal STM (*b* = 0.044, *SE* = 0.094, *t* = 0.472, *p* = .637), verbal WM (*b* = 0.144, *SE* = 0.078, *t* = 1.845, *p* = .066), visuo‐spatial STM (*b* = −0.027, *SE* = 0.106, *t* = −0.255, *p* = .799), and visuo‐spatial WM (*b* = −0.167, *SE* = 0.109, *t* = −1.541, *p* = .124). The effect of age was statistically significant and positive in all models, whereas the effect of sex was not statistically significant (see below). Full model results are reported in Tables S5–S8, and results are plotted in Fig. 3.

The same pattern of results was found when using the total score as the outcome variable, with the effect of age being statistically significant and positive, whereas the effect of sex was not statistically significant (see Tables S9–S12).

##### 3.1.3.3 Effect of age and sex on memory test scores

Significant positive effects of age were found in all models, indicating that older children performed better on memory tasks. Specifically, age was positively related to verbal STM (*b* = 0.344, *SE* = 0.033, *t* = 10.395, *p* < .001), verbal WM (*b* = 0.273, *SE* = 0.028, *t* = 9.906, *p* < .001), visuo‐spatial STM (*b* = 0.306, *SE* = 0.037, *t* = 8.193, *p* < .001), and visuo‐spatial WM (*b* = 0.334, *SE* = 0.038, *t* = 8.747, *p* < .001). Sex was not a significant predictor of memory performance in any task. A similar pattern for the age and sex covariates was observed when using the total score as the outcome variable (see Tables S9–S12).

##### 3.1.3.4 Effects of perceived stress on memory test scores

Models revealed that there was no effect of the PSS‐C on any of the four memory assessments (all *p*’s > .05; see Tables S13–S16).

#### 3.1.4 Discussion

This part of the study examined STM and WM in children from a high‐stress environment (Scampia) and a comparatively lower‐stress setting (Pozzuoli), using conventional verbal and visuo‐spatial tasks. The absence of a “deficit” among children from Scampia, as evidenced by similar scores to those of Pozzuoli across all four tasks, may be attributed to several factors.

One possibility is that Scampia children are not exposed to significant stress; however, in light of our ethnographic work and existing literature on this environment, we find this an unlikely explanation. A more plausible account involves Scampia dense informal support structures—including extended family networks, neighborhood‐based childcare, and long‐standing social bonds—which may serve as protective factors helping sustain STM/WM resilience despite severe structural hardship (see Masten, 2001). This further aligns with cross‐cultural work, suggesting that executive functions do not uniformly decline with socioeconomic disadvantage. For example, Howard et al. (2020) report that early executive functions can be relatively preserved across markedly different socioeconomic contexts, with the impact of stress moderated by children's reactivity to adversity. Similarly, studies involving children in low‐resource contexts have sometimes documented comparable executive function performance to less deprived peers, alongside strengths in other domains such as creativity (e.g., Dahlman, Bäckström, Bohlin, & Frans, 2013; Haft & Hoeft, 2017).

It is also possible that similar performance masks differences in underlying cognitive processes. Neurocognitive research shows that lower‐SES and higher‐SES children can perform equivalently while recruiting different neural systems or compensatory strategies (e.g., D'Angiulli, Herdman, Stapells, & Hertzman, 2008; Moriguchi & Shinohara, 2019). This suggests that developmental conditions may shape *how* cognitive tasks are solved rather than whether they can be solved, pointing to the importance of investigating process‐level differences alongside outcomes.

In addition, testing occurred in familiar school settings rather than in a laboratory. Children from high‐adversity environments can underperform in unfamiliar or evaluative contexts, and reducing such barriers may have allowed their underlying abilities to be more fully expressed (Ellis et al., 2017).

Another explanation derives from the hidden talents model, which suggests that developmental systems may adapt to the demands of harsh environments by prioritizing certain cognitive specializations (Ellis et al., 2017; Frankenhuis & de Weerth, 2013). Navigating Scampia's complexity may create frequent, real‐world demands on short‐term storage and mental manipulation, supporting the development or maintenance of these abilities. At the same time, adversity may calibrate cognitive development in ways not captured by conventional abstract tasks, with other modalities potentially revealing more pronounced developmental differences—a possibility explored in Part 2.

Taken together, these perspectives caution against interpreting the absence of group differences as the absence of environmental influence. Rather, adversity may shape cognitive development in heterogeneous—and sometimes compensatory—ways. Understanding these pathways requires attention not only to outcomes but also to the ecological contexts and cognitive processes through which children engage with everyday challenges.

The PSS‐C aimed to gauge children's perceived stress and anxiety, but scores did not predict memory performance. Based on our ethnographic research and the literature on the study populations, we suggest that the scale did not fully align with the specific stressors these children face, limiting its face validity in these settings. The PSS‐C focuses on general experiences in the past week, such as feeling nervous, sleep difficulties, or relationship with parents, but does not account for the chronic, context‐specific challenges faced by children in Scampia, such as criminal activity. Ethical and cultural constraints precluded the use of more direct questions about sensitive topics (e.g., drugs, violence, food insecurity), which may constitute the most relevant stressors. This raises broader methodological questions about how best to measure adversity and stress in very harsh environments and points to the need for contextually sensitive and ethically acceptable tools.

Overall, Part 1 challenges the assumption that children growing up in high‐stress environments inevitably display cognitive deficits on conventional memory tasks. At the same time, these tasks capture only a subset of children's memory abilities. Research has indicated the existence of additional memory systems for processing different types of information, such as social and motor stimuli (e.g., Meyer et al., 2012; Smyrnis et al., 2014), while other studies have suggested that adversity is associated with the development of enhanced social skills such as better incidental memory for faces (Dietze et al., 2022), increased accuracy in discerning trustworthiness among peers (Pitula et al., 2017), or greater empathic accuracy (Kraus et al., 2010). This underscores the pressing need for examining more ecologically relevant memory modalities, a question directly addressed in Part 2.

### 3.2 Part 2—Social memory

Building upon Part 1, which utilized conventional, abstract stimuli in assessing memory capacities, Part 2 was designed to explore the response to social stimuli in STM and WM in the same cohort of children.

#### 3.2.1 Method

##### 3.2.1.1 Participants

The same 357 children recruited for Part 1 also participated in Part 2, and recruitment procedures and exclusion criteria remained identical. Children younger than 5 years old (*n* = 16), older than 11 years (*n* = 1), and those who did not understand the tasks (*n*_social STM_ = 2, *n*_social WM_ = 3) were excluded from the analyses. The refined sample sizes are reported in Table S17; exclusions by test are detailed in Table S18.

##### 3.2.1.2 Procedure

This part of the study included two tasks assessing social STM and social WM, following the same procedure as Part 1.

##### 3.2.1.3 Measures

To assess social STM and WM, we adapted the visuo‐spatial memory task from Part 1, substituting conventional, abstract stimuli with social stimuli. These consisted of high‐quality photos of children's faces, retrieved from an online free archive (www.pexels.com). The selection included 136 images of children from diverse ethnic backgrounds, with an equal sex balance. The depicted children ranged in age from approximately 6 to 10 years old, aligning with the study participants’ age group. The facial expressions varied from neutral to positive. All photos were cropped to a uniform dimension and printed on laminated A5 cards (14.8 cm × 21 cm).

In the social STM task, participants were required to point with their finger to the face cards in the same order as presented. Face cards were shown one at a time for 1 second each, positioned at the child's eye level at about 80 cm. After the presentation, the cards were placed on the table following the pattern of the visuo‐spatial task of Part 1. The comprehension check followed the same procedure as Part 1.

The task began with two different lists of two items, and both were administered. If the child correctly recalled at least one list, they progressed to longer ones (increased by one item per iteration). The task continued until the child correctly recalled at least one list, with correct recall requiring the reproduction of all elements in the presented order. To assess social WM, a similar task was used, but with children asked to recall the items in reverse order. As in Part 1, the tasks generate two scores: the *span* score indicates the length of the longest correctly recalled list, while a *total* score was calculated as the sum of all correctly recalled lists. Our main analyses focused on the span score, and analyses of total scores are reported in full in the Supplementary Material.

#### 3.2.2 Analyses

We ran two linear regression models, one for each of the dependent variables (i.e., social STM and social WM). The main predictor variable was participant environment, and we included participant age and sex as covariates. Treatment contrasts were applied on the environment variable, using Pozzuoli (the lower‐stress population) as the reference category. Additionally, we tested whether participants’ PSS‐C scores predicted the dependent variables using linear models. We did not exclude outlying observations. Participant data were only excluded if they were unable to follow or complete the memory task (see Table S18).

#### 3.2.3 Results

##### 3.2.3.1 Descriptive statistics

Task comprehension was high overall (see Table S19), and scores are reported in Table 3.

**Table 3:** Span scores for social STM and WM tasks across the two populations

Environment	Social STM		Social WM	
	*M*	*SD*	*M*	*SD*
Pozzuoli	5.27	1.47	4.48	1.73
Scampia	6.11	1.39	4.94	1.66

The PSS‐C data from Part 1 were used to test the effects of perceived stress on social memory performance.

##### 3.2.3.2 Effects of environment on memory test scores

**Fig. 4:**
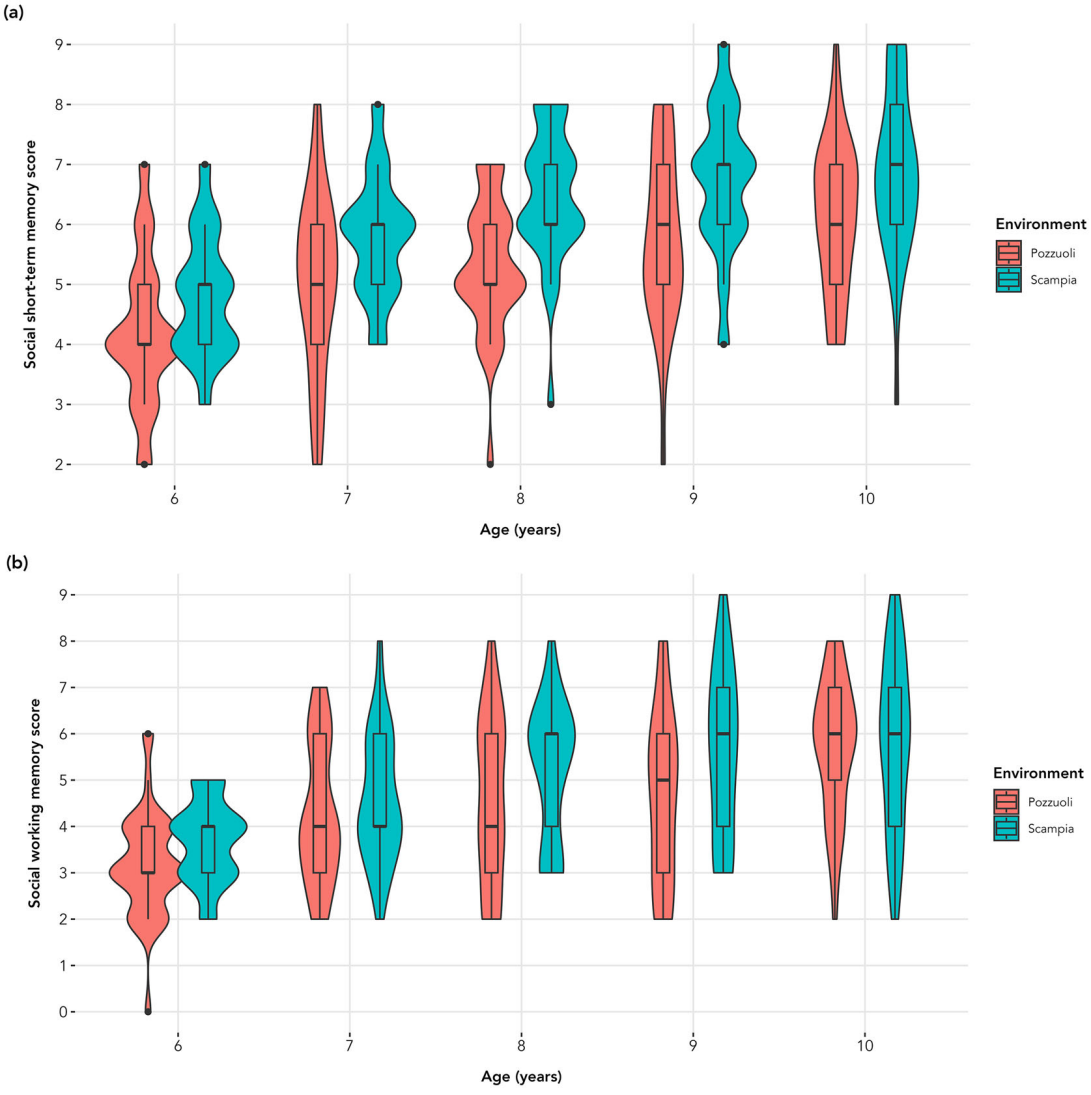
Participants’ social STM (panel a) and social WM (panel b) span scores by environment and age.

Scampia children scored significantly higher than those from Pozzuoli in both social STM (*b* = 0.835, *SE* = 0.137, *t* = 6.087, *p* < .001) and social WM (*b* = 0.460, *SE* = 0.168, *t* = 2.743, *p* = .006). Full model results are reported in Tables S20 and S21, and results are plotted in Fig. 4.

When employing the total score as the outcome variable, the same pattern of results was observed (see Tables S22 and S23).

##### 3.2.3.3 Effect of age and sex on memory test scores

Significant positive effects of age were found in all models, indicating that older children had better performance on memory tasks. Specifically, age was positively related to social STM (*b* = 0.489, *SE* = 0.048, *t* = 10.131, *p* < .001) and social WM (*b* = 0.526, *SE* = 0.059, *t* = 8.924, *p* < .001).

Sex was not a significant predictor of memory performance on either social STM task (*b* = −0.051, *SE* = 0.137, *t* = −0.374, *p* = .709) or social WM task (*b* = 0.171, *SE* = 0.167, *t* = 1.022, *p* = .307).

##### 3.2.3.4 Effects of perceived stress on memory test scores

Results suggest no effect of the PSS‐C on either assessment of memory (both *p*’s > .05; see Tables S24 and S25).

#### 3.2.4 Discussion

Part 2 examined STM and WM using social stimuli with children from different environments in the Naples metropolitan area. Children from Scampia performed significantly better than their Pozzuoli peers in both social STM and social WM. For example, the 7‐year‐olds from Scampia scored comparably to the 9‐year‐olds from Pozzuoli (see Fig. 4). This suggests that children from more stressful environments may have more advanced memory abilities for socially relevant information earlier in development.

From an evolutionary‐developmental perspective, natural selection has shaped developmental systems to adjust to environmental demands (Bjorklund & Ellis, 2014), and memory can be considered an adaptive function that evolved to prioritize fitness‐relevant information (see Nairne, Pandeirada, Gregory, & Van Arsdall, 2009). Consistent with this view, previous research has demonstrated a propensity for children to acquire and remember information about potential threats, such as dangerous animals (Barrett & Broesch, 2012) or negative social actions (Baltazar, Shutts, & Kinzler, 2012). In Scampia, where social competition and criminal activity are relatively prevalent, threats primarily originate from fellow humans. Navigating this environment requires being constantly vigilant, detect potential threats, recognize individuals from different factions, distinguish law enforcement officers, and understand social hierarchies and appropriate behaviors. Our results suggest that children in Scampia have developed enhanced social memory as an adaptation to this challenging social landscape.

Notably, this pattern is not confined to the Neapolitan context: a recent replication conducted with children in rural and urban Mozambique revealed a similar advantage in social STM and WM among children from the higher‐stress environment (Paone, Davis, & Cohen, 2026), suggesting that enhanced performance in socially meaningful memory tasks may reflect broader patterns of development under adversity.

These findings also align with recent work comparing performance on abstract versus ecologically relevant cognitive tasks among adversity‐exposed individuals. In three studies—Frankenhuis et al. (2020), Rifkin‐Graboi et al. (2021), and Young, Frankenhuis, DelPriore, and Ellis (2022)—participants who had been exposed to adversity performed more poorly on abstract tasks but showed equal or superior performance when tested with ecologically meaningful stimuli. However, domains of enhancement differed across studies (e.g., social dominance reasoning, socioemotional memory, WM updating), suggesting that adversity does not produce a single, uniform cognitive profile but rather domain‐specific specializations shaped by local demands. This convergence strengthens the interpretation that conventional STM/WM tasks may underestimate relevant cognitive abilities in children from harsh environments, while ecologically grounded measures could reveal competencies that are otherwise obscured. At the same time, some divergence across studies highlights important open questions, such as which environmental features select for particular skills, and why enhancements appear in some domains but not others.

As in Part 1, perceived stress as measured by the PSS‐C did not correlate with performance on social memory tasks. This suggests that factors influencing memory performance in high‐stress environments may be more complex than can be captured by self‐reported stress measures alone (see Part 1 discussion).

Overall, Part 2 provides evidence that harsh environments can cultivate adaptive cognitive skills in STM and WM. These findings also highlight the necessity of using ecologically relevant tasks when researching with children from diverse environments: the stronger performance of the Scampia sample would have remained “hidden” if only conventional memory tasks had been used, as in Part 1.

**Fig. 5:**
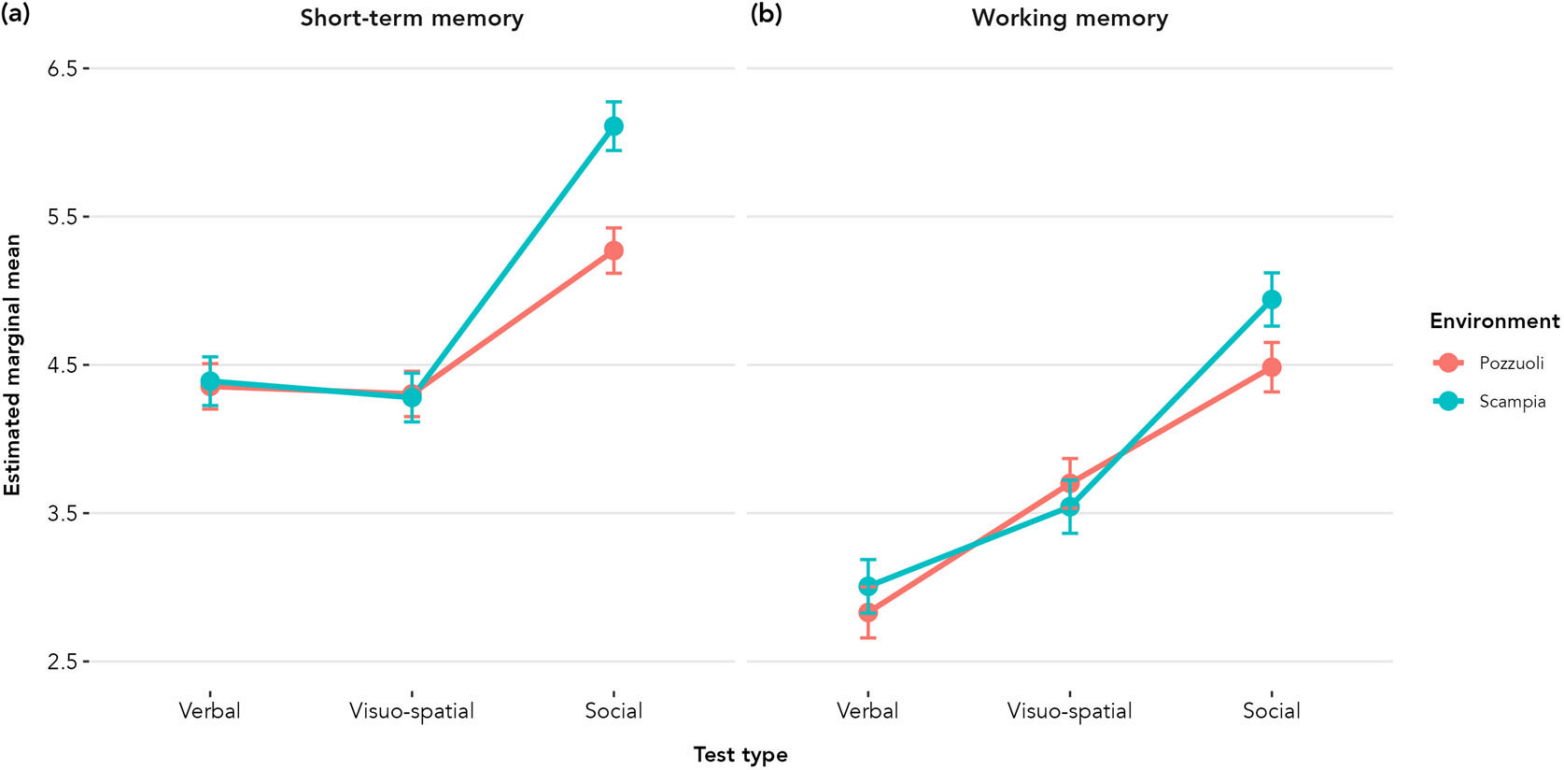
Memory performance across task types and environments. Estimated marginal means for STM (panel a) and WM (panel b) across verbal, visuo‐spatial, and social tasks for children from Pozzuoli and Scampia. Points represent model‐estimated marginal means adjusted for age and sex; error bars indicate 95% confidence intervals.

To facilitate comparison of performance across memory modalities and environments, Fig. 5 presents the estimated marginal mean for verbal, visuo‐spatial, and social STM and WM tasks, calculated using multilevel models with test scores as the outcome variable, the environment by test type interaction as the main predictor, sex and age as covariates, and participant as the level‐two grouping variable.

### 3.3 Comparison of environment effect sizes by test type

Parts 1 and 2 examined the effects of environment on STM and WM using abstract and social stimuli, respectively. While these analyses establish whether performance varies across environments within each task type, they do not allow for comparisons of relative differences in effect sizes by test type. To investigate this, we calculated and compared effect sizes for each task in both parts of the study using Cohen's *d* with bootstrapped confidence intervals.

#### 3.3.1 Cohen's *d* analysis

Using bootstrap confidence intervals for Cohen's *d*, we compared the effect sizes of environment (Scampia vs. Pozzuoli) for each of the six test types across Part 1 and Part 2. Confidence intervals were calculated using the BootES package in R (Kirby & Gerlanc, 2013), with each comparison based on 10,000 resamples. Results and summary statistics are shown in Fig. S1.

Differences between Scampia and Pozzuoli children were not statistically significant for verbal STM, verbal WM, visuo‐spatial STM, and visuo‐spatial WM. However, in social STM (*d =* 0.597, 95% CI = [0.384, 0.819]) and social WM (*d* = 0.272, 95% CI = [0.057, 0.484]), Scampia children scored significantly higher than their Pozzuoli peers, indicating relatively stronger performance in these tasks for Scampia children, especially in social STM (see Table S26 for full details).

## 4 General discussion

This study examined STM and WM in children growing up in markedly different environments, using both conventional abstract tasks and ecologically relevant social tasks. The findings revealed that children from Scampia (a high‐stress environment) performed comparably to peers from Pozzuoli (a lower‐stress environment) on abstract STM and WM tasks, and outperformed them on social STM and WM tasks, with some age groups scoring comparably to Pozzuoli children up to 2 years older. These results underscore that cognitive performance under adversity cannot be adequately characterized by a single dimension of “impairment,” but instead depends critically on the nature of the cognitive demands and the relevance of the information being processed.

Our study contributes to an emerging line of research that has compared cognitive performance under adversity using both conventional abstract tasks and more ecologically relevant ones. Frankenhuis et al. (2020) showed that adolescents and adults exposed to violence exhibited enhanced memory and reasoning about social dominance, a domain in which errors can carry substantial costs. Rifkin‐Graboi et al. (2021) found that early caregiving adversity was associated with enhanced memory for emotionally salient social information, suggesting accelerated or prioritized development of systems involved in managing interpersonal threat. Young et al. (2022) demonstrated that adversity‐exposed youth showed equivalent performance on WM updating tasks when these involved ecologically relevant stimuli, despite poorer performance on abstract versions of the same tasks.

The present findings extend this emerging pattern in several important ways. First, they demonstrate such modality‐specific differences in middle childhood, a developmental period that has been underrepresented in this literature. Second, they show that these effects are evident not only in higher‐order reasoning or updating, but also in core memory systems, specifically STM and WM capacity. Third, unlike some prior studies that report trade‐offs between abstract and ecological performance, we observed no abstract‐task deficit among children from Scampia. This suggests that adaptation to adversity does not necessarily entail reduced capacity in conventionally assessed domains, highlighting the heterogeneity of developmental pathways under adversity and cautioning against assuming a uniform pattern of impairments and enhancements.

One interpretation of the absence of an abstract‐memory deficit in Scampia children is that protective factors buffer development against the negative effects of chronic adversity. Despite severe structural hardship, Scampia is characterized by dense informal support structures, including extended family networks, neighborhood‐based childcare, and strong social ties, factors that can moderate the effects of stress on development and contribute to resilience (Masten & Barnes, 2018). At the same time, the present findings also point beyond traditional resilience models. Resilience frameworks typically emphasize normative development *despite* adversity. In contrast, the superior performance of Scampia children on social memory tasks suggests the possibility of calibrated cognitive development—skills that emerge *because of* the demands imposed by the environment. Scampia is characterized by intense social competition and widespread criminal activity, conditions under which accurately tracking individuals, alliances, and potential threats carries heightened adaptive value. This interpretation aligns with the hidden talents model, which proposes that developmental systems allocate resources toward abilities that are most useful for solving recurrent problems in harsh and unpredictable contexts (Ellis et al., 2022; Frankenhuis & de Weerth, 2013).

Furthermore, our results also align with theories suggesting that stressful environments may foster the development of cooperative strategies (e.g., Ember et al., 2018; Whitehouse et al., 2017). Developing superior memory for individual recognition could be particularly advantageous in resource‐scarce environments, helping children identify who to trust, avoid, or cooperate with. This also connects with theories of tight‐loose societies (e.g., Gelfand et al., 2006). In tighter environments such as Scampia, where criminal networks operate under strict social norms, there are severe consequences for norm violations, and enhanced social memory could be particularly beneficial for monitoring others’ behavior, navigating social hierarchies, avoiding conflict, and forming strategic alliances.

Importantly, these perspectives are not mutually exclusive. Comparable performance on abstract tasks may reflect buffering processes that sustain core cognitive capacities, while enhanced performance in socially relevant tasks may reflect adaptive specialization tuned to environmental demands. The present results, therefore, suggest that development under adversity involves a combination of preserved functioning, compensatory processes, and domain‐specific enhancement.

A corollary of the specialization hypothesis within the hidden talents model is the *sensitization hypothesis* (Ellis et al., 2017), which suggests that stress‐adapted skills can find optimal expression in conditions resembling those in which they were originally acquired. From this view, cognitive, physical, and behavioral skills honed under conditions of adversity may remain hidden in certain contexts but become readily observed in others that mirror the demands of that environment. For example, (combat) sports require constant vigilance, rapid decision‐making, and the ability to anticipate others’ actions, skills that may be highly adaptive in both sport and in daily life within high‐stress environments, but may be less apparent in a school classroom setting. Notably, the Scampia neighborhood has produced a remarkable number of elite judo athletes, including multiple European and world champions as well as an Olympic gold medalist. While many interacting factors undoubtedly contribute to this pattern, it is plausible that locally adaptive skills developed under conditions of adversity may find particularly strong expression in contexts that share similar demands.

At a broader theoretical level, our findings contribute to ongoing debates about the nature of executive functions and cognitive abilities more generally. Much of the executive‐function literature implicitly treats these abilities as stable, context‐independent capacities that reside within individuals and can be measured through decontextualized tasks. However, an increasing body of work argues that executive functions are inherently goal‐ and context‐dependent, reflecting interactions between individuals, tasks, and environments (e.g., Doebel, 2020). From this perspective, STM and WM are not merely internal storage systems but tools for accomplishing contextually meaningful goals. The present findings are consistent with this view. Abstract and social memory tasks place different demands on attention, motivation, and information processing, and they likely engage cognitive resources in different ways. Superior performance on social memory tasks among Scampia children suggests that executive resources may be more readily deployed when tasks align with everyday informational priorities such as tracking identities, intentions, alliances, and potential threats in complex social environments.

These results also resonate with broader approaches in cognitive science that conceptualize cognition as situated, embodied, and environmentally scaffolded (Clark, 1996; Hutchins, 1995; Newen, Bruin, & Gallagher, 2018). Cognitive processes emerge through ongoing interactions between minds, bodies, and the surrounding environments, rather than residing solely within the individual's brain. In this light, modality‐specific memory performance may reflect not fixed cognitive capacities, but the alignment—or misalignment—between task demands and the cognitive practices cultivated through everyday experience.

Our findings also contribute to broader discussions of intelligence as adaptive functioning. If intelligence is defined narrowly as performance on context‐free cognitive tests, then the observed social‐memory advantage may not qualify as “intelligence.” However, if intelligence is understood as the capacity to solve problems that matter within one's lived environment (Ellis et al., 2023), then enhanced social memory in Scampia children can be viewed as a form of adaptive cognitive competence, consistent with cross‐cultural evidence showing that forms of adaptive knowledge are often tailored to local ecological demands (Grigorenko et al., 2004; Paone, 2025; Sternberg et al., 2001).

The mechanisms underlying these patterns remain an open question. One possibility is that repeated exposure to socially salient and potentially threatening situations enhances attention to, encoding of, and retention of social information. Prior work suggests that adversity heightens attentional engagement with emotionally or socially salient stimuli, which in turn can facilitate memory formation (Dietze et al., 2022; Rifkin‐Graboi et al., 2018). Another possibility is that children from high‐stress environments recruit cognitive resources differently, even when behavioral performance appears similar. Neurocognitive studies have shown that children from different socioeconomic backgrounds can achieve comparable task performance while engaging distinct neural systems or compensatory strategies (D'Angiulli et al., 2008; Moriguchi & Shinohara, 2019). Although the present study did not include neural measures, these findings raise the possibility that similar abstract‐task performance may mask differences in underlying cognitive organization, while social tasks more clearly reveal environmentally shaped specialization. Longitudinal and neuroimaging research will be essential for clarifying how early adversity shapes the development and recruitment of memory‐related neural systems over time.

A key implication of this work concerns how cognitive abilities are assessed. Had we relied exclusively on conventional abstract tasks, we would have concluded that children from Scampia do not differ meaningfully from those in Pozzuoli, and the social‐memory advantage observed in Part 2 would have remained invisible. This raises questions about fairness and validity in measurement: treating all individuals identically by administering the same abstract tasks may achieve methodological equality, but it does not necessarily yield conceptual equity—providing individuals with opportunities to demonstrate the skills that matter for functioning in *their* environments. Incorporating ecologically relevant tasks can, therefore, enrich our understanding of cognition while also addressing long‐standing concerns about the representativeness and ecological validity of psychological research.

For a finer‐grained analysis of how environmental context shapes memory performance, we compared effect sizes across different test types. Cohen's *d* analysis revealed that Scampia children performed better than Pozzuoli children in social STM and WM tasks, showing especially strong (relative) performance in social STM (*d* = 0.600). Rapid encoding and retention of socially relevant information may be especially adaptive in environments characterized by social threat and competition, whereas WM additionally relies on controlled manipulation processes that are more strongly influenced by formal schooling, potentially narrowing environmental differences.

It is important to recognize that environmental conditions may differentially affect STM and WM, and further research is needed into how these systems are shaped by specific environmental pressures to support adaptation to diverse survival demands, particularly in underrepresented populations. The absence of a significant relationship between perceived stress levels as measured by the PSS‐C and memory performance potentially reflects the complexity of defining and measuring exposure to adversity in young children. For ethical and other reasons, the PSS‐C and similar tools likely do not adequately capture the types and severity of stressors faced by children in extremely harsh environments. Future research should aim to develop more ecologically valid measures and interventions that can accurately reflect the specific stressors and coping mechanisms relevant to children in these contexts.

## 5 Contributions and limitations

This research holds several notable strengths. First, it focuses on young children aged 6–10, diverging from most memory studies that primarily focus on adults. Second, to the best of our knowledge, this is the first study to systematically examine STM and WM for social information in children growing up in a high‐stress urban environment and to directly compare performance on abstract versus ecologically relevant memory tasks within the same population. Third, we collected data from a large sample exceeding 350 children across two contrasting environments, including a resource‐poor and high‐stress one. Our findings thus help address the strong bias toward student‐based W.E.I.R.D. participants in research (Henrich et al., 2010), and demonstrate the feasibility of conducting rigorous developmental studies in settings that are often considered logistically and ethically challenging.

A further strength lies in the integration of complementary methodologies. Standardized cognitive assessments were combined with extended ethnographic fieldwork, an approach that allows cognitive performance to be interpreted in light of children's lived environments rather than as isolated test outcomes.

Furthermore, integrating cognitive and ethnographic research provides a comprehensive perspective on the investigated skills and the environments influencing their development. In addition, by pairing conventional abstract tasks with novel tasks designed to be more ecologically relevant, the study illustrates how conclusions about cognitive development can vary substantially depending on the ecological relevance of the assessment tools. In doing so, it challenges deficit‐based interpretations that rely exclusively on decontextualized measures and highlights how certain cognitive strengths may remain hidden when inappropriate tasks are used. Results could help tailor education to account for the specific memory development profiles of the groups of children tested, potentially leading to more effective educational approaches. Additionally, this research challenges stereotypes often associated with low‐SES backgrounds since individuals from such environments frequently face stigmatization, which can significantly impact their social identity and opportunities (Cozzarelli, Wilkinson, & Tagler, 2001; Spencer & Castano, 2007).

A limitation concerns the assessment of children's exposure to environmental stressors. We relied on the PSS‐C to capture children's subjective experience of stress and anxiety. Self‐report measures of stress in childhood are known to be influenced by developmental constraints (Allwood, Gaffey, Vergara‐Lopez, & Stroud, 2017). Children may also show response biases in questionnaire formats, such as selecting extreme responses, referring to a single event, or exhibiting reluctance to recall negative past experiences (Rotsika et al., 2011; Varni, Limbers, & Burwinkle, 2007). Acquiring reliable questionnaire responses from parents is equally challenging, as they may not want to disclose certain information, or misperceive their child's stress levels, potentially exaggerating difficulties (see Allwood et al., 2017; Rotsika et al., 2011). The PSS‐C captures recent, perceived stress rather than chronic, structural, or context‐specific adversity. In environments characterized by persistent hardship, exposure to violence, or institutional neglect, children may normalize stressors that would be considered exceptional in safer contexts, reducing the correspondence between subjective stress reports and objective environmental conditions. At the same time, direct assessment of many stressors commonly used as proxies for adversity (e.g., neighborhood violence, caregiver instability, illegal activities) was not feasible for ethical, cultural, and safety reasons, particularly given the young age of participants and the sensitivity of the research settings. To mitigate these limitations, we complemented the PSS‐C with environmental indices measuring social and material vulnerability, alongside ethnographic data.

Another consideration is the impact of the COVID‐19 pandemic on the children's irregular school attendance during the 2 years preceding data collection, which could have affected their performance. Several studies have demonstrated a correlation between schooling and outcomes on STM and WM tasks (e.g., Kosmidis, Zafiri, & Politimou, 2011; Roberts et al., 2015). However, children from Scampia and Pozzuoli attended schools within the same municipal area and were, therefore, subject to the same COVID‐19 regulations, including school closures and reopenings, ensuring comparable exposure to pandemic‐related educational disruptions across sites.

Furthermore, as a natural experiment, diverse environments pose challenges with potential confounding factors. While stress adaptation may explain some variation, unaccounted factors may also contribute to observed differences.

## 6 Conclusion

This research provides new evidence that memory development in children raised in adverse environments cannot be adequately characterized by a simple deficit model. Using both abstract and socially meaningful tasks, we showed that children from a high‐stress urban environment performed comparably to peers from a lower‐stress setting on conventional STM and WM measures, while demonstrating superior performance on social memory tasks. These findings show the context‐dependent nature of cognitive development and illustrate how conclusions about impairment or competence depend critically on what is measured and how.

More broadly, the results support emerging perspectives in cognitive and behavioral science that emphasize adaptation, calibration, and ecological validity. Cognitive abilities do not develop in a vacuum but are shaped by the demands, risks, and opportunities of children's everyday environments. When assessments fail to reflect those factors, important skills may remain invisible. By contrast, ecologically relevant tasks, alongside ethnographic research, can reveal forms of cognitive competence that are directly aligned with the challenges children face in their daily lives.

Recognizing these “hidden talents” has implications beyond theory. It calls for greater attention to equity in cognitive assessment and education—not merely treating all children identically, but providing opportunities for diverse skills to be recognized, supported, and leveraged. Future studies should continue to integrate cognitive testing with ethnographic research, expand the range of ecologically relevant tasks, and explore the neural and developmental mechanisms through which adversity shapes cognition. Such efforts are essential for building a more inclusive and accurate science of human development.

## Funding

This research was funded by the Economic and Social Research Council Economic and Social Research Council grant ES/P000649/1, by St John's College of the University of Oxford, and by the School of Anthropology and Museum Ethnography of the University of Oxford.

## Conflict of interest

The authors have no known conflict of interest to disclose.

## Ethics approval statement

This research was approved by the relevant Departmental Research Ethics Committee of the University of Oxford (Ref No.: SAME_C1A_22_021).

## Permission to reproduce material from other sources

All material in this manuscript is original, and any reproduced material has been appropriately cited or is within the public domain.

## Supporting information



Supporting Information

## Data Availability

The data, analysis code, and study materials supporting the findings of this study are publicly available on our Open Science Framework (OSF) project website at: https://osf.io/yuzcd/.
