# Supplementary material for: Modality Matters: Intact and Enhanced Memory Skills in Children From High‐Stress Environments
Source: Cogn Sci. 2026 Apr 29;50:e70214. doi: 10.1111/cogs.70214 (PMC13128153; doi:10.1111/cogs.70214)
Supplement: Supplementary file 1 — Supporting Information [file COGS-50-e70214-s001.docx]

**Supplementary material**

1. **Indice di vulnerabilità sociale e materiale (IVSM, Istat)**

The IVSM is a composite indicator developed by the Italian National Institute of Statistics (Istat). It is constructed from seven indicators derived primarily from the 2011 Population and Housing Census data, the last traditional decennial census conducted in Italy. The IVSM captures multiple dimensions of social and material vulnerability, including educational attainment, family size, single-parent demographics, caregiving burden, housing overcrowding, youth inactivity, and economic hardship. The methodological description of the index is provided in an Istat report published in 2020, which also presents updated territorial analyses.

1. **Power analyses**

For Study 1, sample sizes were determined by a power analysis, which assumed a power of 0.8, and that children from high-stress environments would have verbal memory test span scores on average 0.5 lower than children from Pozzuoli and visuospatial memory test span scores on average 0.3 lower than children from Pozzuoli. The power analyses suggested a sample size of 125 participants per environment to give a power of at least 0.8 to detect all effect sizes. The full power analysis can be found here: https://osf.io/yuzcd/.

For Study 2, sample sizes were determined by a power analysis that was based on piloting and data from conventional working memory tests. The power analysis showed that 125 participants per environment would be needed to have a power of 0.84 to detect a 0.4-point difference in visuospatial social memory test span scores between the higher-stress environments of Scampia and the lower-stress environment of Pozzuoli (i.e., a positive effect of higher-stress environment on visuospatial social memory test span scores). The power analysis used an alpha (significance) level of 0.05 for its hypothesis testing. The full power analysis can be found here: https://osf.io/yuzcd/.

1. **Tables**

**Part 1**

**Table 1.** Participants excluded from analysis; reason for exclusion by environment.

| **Test** | **≤ 5 years old** | **≥ 11 years old** | **Did not understand** |  | **Total** |
| --- | --- | --- | --- | --- | --- |
| Verbal STM | 16 (S: 8, P: 8) | 1 (S: 1) | 3 (S: 1, P: 2) |  | 20 |
| Verbal WM | 16 (S: 8, P: 8) | 1 (S: 1) | 17 (S: 4, P: 13) |  | 34 |
| Visuo-spatial STM | 16 (S: 8, P: 8) | 1 (S: 1) | 3 (S: 2, P: 1) |  | 20 |
| Visuo-spatial WM | 16 (S: 8, P: 8) | 1 (S: 1) | 5 (S: 2, P: 3) |  | 22 |

**Table 2.** Children’s tasks understanding.

| **Sample** | **Verbal STM** | **Verbal WM** | **Visuo-spatial STM** | **Visuo-spatial WM** |
| --- | --- | --- | --- | --- |
| Scampia | 99.37% | 97.47% | 98.73% | 98.73% |
| Pozzuoli | 98.90% | 92.86% | 99.45% | 98.35% |

**Table 3.** One-Way ANOVA Results for Perceived Stress Scale for Children (PSS-C) Scores by Environment.

| **Source** | ***df*** | **Sum of Squares** | **Mean Square** | ***F*** | ***p*** |
| --- | --- | --- | --- | --- | --- |
| **Environment** | 1 | 53 | 52.77 | 2.56 | .111 |
| **Residual** | 337 | 6950 | 20.62 |  |  |

**Table 4.** Tukey HSD Post-hoc Comparisons for PSS-C Scores by Environment.

| **Comparison** | **Mean Difference** | **95% CI Lower** | **95% CI Upper** | ***p*-adj** |
| --- | --- | --- | --- | --- |
| **Scampia – Pozzuoli (S–P)** | 0.79 | -0.182 | 1.764 | .111 |

**Table 5.** Regression model for effect of environment on span scores for verbal STM.

| **Variable** | ***b*** | ***SE*** | ***t*** | ***p*** |
| --- | --- | --- | --- | --- |
| **Environment (Scampia)** | 0.044 | 0.094 | 0.472 | .637 |
| **Sex (Male)** | 0.134 | 0.093 | 1.429 | .154 |
| **Age** | 0.344 | 0.033 | 10.395 | < .001 |
| Multiple *R^2^* = 0.250, Adjusted R-squared = 0.243 | | | | |

**Table 6.** Regression model for effect of environment on span scores for verbal WM.

| **Variable** | ***b*** | ***SE*** | ***t*** | ***p*** |
| --- | --- | --- | --- | --- |
| **Environment (Scampia)** | 0.144 | 0.078 | 1.845 | .066 |
| **Sex (Male)** | 0.074 | 0.078 | 0.946 | .345 |
| **Age** | 0.273 | 0.028 | 9.906 | < .001 |
| Multiple *R^2^* = 0.242, Adjusted R-squared = 0.235 | | | | |

**Table 7.** Regression model for effect of environment on span scores for visuo-spatial STM.

| **Variable** | ***b*** | ***SE*** | ***t*** | ***p*** |
| --- | --- | --- | --- | --- |
| **Environment (Scampia)** | −0.027 | 0.106 | −0.255 | .799 |
| **Sex (Male)** | −0.147 | 0.106 | −1.386 | .167 |
| **Age** | 0.306 | 0.037 | 8.193 | < .001 |
| Multiple *R^2^* = 0.171, Adjusted R-squared = 0.164 | | | | |

**Table 8.** Regression model for effect of environment on span scores for visuo-spatial WM.

| **Variable** | ***b*** | ***SE*** | ***t*** | ***p*** |
| --- | --- | --- | --- | --- |
| **Environment (Scampia)** | −0.167 | 0.109 | −1.541 | .124 |
| **Sex (Male)** | −0.040 | 0.108 | −0.370 | .712 |
| **Age** | 0.334 | 0.038 | 8.747 | < .001 |
| Multiple *R^2^* = 0.192, Adjusted R-squared = 0.185 | | | | |

**Table 9.** Regression model for effect of environment on total scores for verbal STM.

| **Variable** | ***b*** | ***SE*** | ***t*** | ***p*** |
| --- | --- | --- | --- | --- |
| **Environment (Scampia)** | 0.457 | 0.761 | 0.601 | .548 |
| **Sex (Male)** | 0.545 | 0.759 | 0.718 | .473 |
| **Age** | 3.147 | 0.268 | 11.723 | < .001 |
| Multiple *R^2^* = 0.294, Adjusted R-squared = 0.288 | | | | |

**Table 10.** Regression model for effect of environment on total scores for verbal WM.

| **Variable** | ***b*** | ***SE*** | ***t*** | ***p*** |
| --- | --- | --- | --- | --- |
| **Environment (Scampia)** | 0.431 | 0.419 | 1.029 | .304 |
| **Sex (Male)** | 0.318 | 0.418 | 0.761 | .447 |
| **Age** | 1.626 | 0.148 | 10.993 | < .001 |
| Multiple *R^2^* = 0.277, Adjusted R-squared = 0.270 | | | | |

**Table 11.** Regression model for effect of environment on total scores for visuo-spatial STM.

| **Variable** | ***b*** | ***SE*** | ***t*** | ***p*** |
| --- | --- | --- | --- | --- |
| **Environment (Scampia)** | −0.153 | 0.795 | −0.192 | .848 |
| **Sex (Male)** | −1.078 | 0.793 | −1.360 | .175 |
| **Age** | 2.478 | 0.280 | 8.864 | < .001 |
| Multiple *R^2^* = 0.194, Adjusted R-squared = 0.187 | | | | |

**Table 12.** Regression model for effect of environment on total scores for visuo-spatial WM.

| **Variable** | ***b*** | ***SE*** | ***t*** | ***p*** |
| --- | --- | --- | --- | --- |
| **Environment (Scampia)** | −0.848 | 0.704 | −1.205 | .229 |
| **Sex (Male)** | 0.226 | 0.702 | 0.322 | .747 |
| **Age** | 2.368 | 0.247 | 9.575 | < .001 |
| Multiple *R^2^* = 0.220, Adjusted R-squared = 0.213 | | | | |

**Table 13.** Regression model for effect of PSS on span scores for verbal STM.

| **Variable** | ***b*** | ***SE*** | ***t*** | ***p*** |
| --- | --- | --- | --- | --- |
| **Intercept** | 1.555 | 0.294 | 5.283 | < .001 |
| **PSS** | −0.014 | 0.010 | −1.390 | .166 |
| **Sex (Male)** | 0.127 | 0.093 | 1.363 | .174 |
| **Age** | 0.348 | 0.033 | 10.510 | < .001 |
| Multiple *R^2^* = 0.253, Adjusted R-squared = 0.247 | | | | |

**Table 14.** Regression model for effect of PSS on span scores for verbal WM.

| **Variable** | ***b*** | ***SE*** | ***t*** | ***p*** |
| --- | --- | --- | --- | --- |
| **Intercept** | 0.587 | 0.248 | 2.371 | .018 |
| **PSS** | 0.005 | 0.009 | 0.575 | .566 |
| **Sex (Male)** | 0.065 | 0.078 | 0.832 | .406 |
| **Age** | 0.271 | 0.028 | 9.707 | < .001 |
| Multiple *R^2^* = 0.235, Adjusted R-squared = 0.228 | | | | |

**Table 15.** Regression model for effect of PSS on span scores for visuo-spatial STM.

| **Variable** | ***b*** | ***SE*** | ***t*** | ***p*** |
| --- | --- | --- | --- | --- |
| **Intercept** | 1.722 | 0.333 | 5.168 | < .001 |
| **PSS** | 0.011 | 0.012 | 0.970 | .333 |
| **Sex (Male)** | −0.141 | 0.106 | −1.333 | .183 |
| **Age** | 0.302 | 0.037 | 8.071 | < .001 |
| Multiple *R^2^* = 0.173, Adjusted R-squared = 0.166 | | | | |

**Table 16.** Regression model for effect of PSS on span scores for visuo-spatial WM.

| **Variable** | ***b*** | ***SE*** | ***t*** | ***p*** |
| --- | --- | --- | --- | --- |
| **Intercept** | 0.867 | 0.342 | 2.534 | 0.012 |
| **PSS** | −0.001 | 0.012 | −0.098 | .922 |
| **Sex (Male)** | −0.030 | 0.109 | −0.276 | .783 |
| **Age** | 0.334 | 0.038 | 8.678 | < .001 |
| Multiple *R^2^* = 0.186, Adjusted R-squared = 0.179 | | | | |

**Part 2**

**Table 17.** Demographic information of participants across tasks.

| **Environment** | **Task type** | ***n*** | ***n_females_* (%)** | ***M_age_* (years)** | ***SD_age_* (years)** |
| --- | --- | --- | --- | --- | --- |
| Scampia | Social STM | 157 | 83 (52.9) | 8.35 | 1.41 |
|  | Social WM | 156 | 83 (53.2) | 8.35 | 1.42 |
| Pozzuoli | Social STM | 181 | 84 (46.41) | 8.33 | 1.42 |
|  | Social WM | 181 | 84 (46.41) | 8.33 | 1.42 |

**Table 18.** Participants excluded from analysis (reason for exclusion and total).

| **Test** | **≤ 5 years old** | **≥ 11 years old** | **Did not understand** | **Total** |
| --- | --- | --- | --- | --- |
| Social STM | 16 (S: 8, P: 8) | 1 (S: 1) | 2 (S: 1, P: 1) | 19 |
| Social WM | 16 (S: 8, P: 8) | 1 (S: 1) | 3 (S: 2, P: 1) | 20 |

**Table 19.** Children’s tasks understanding.

| **Sample** | **Social STM** | **Social WM** |
| --- | --- | --- |
| Scampia | 99.37% | 99.73% |
| Pozzuoli | 99.45% | 99.45% |

**Table 20.** Regression model for effect of environment on span scores for social STM.

| **Variable** | ***b*** | ***SE*** | ***t*** | ***p*** |
| --- | --- | --- | --- | --- |
| **Environment (Scampia)** | 0.835 | 0.137 | 6.087 | < .001 |
| **Sex (Male)** | −0.051 | 0.137 | −0.374 | .709 |
| **Age** | 0.489 | 0.048 | 10.131 | < .001 |
| Multiple *R^2^* = 0.297, Adjusted R-squared = 0.291 | | | | |

**Table 21.** Regression model for effect of environment on span scores for social WM.

| **Variable** | ***b*** | ***SE*** | ***t*** | ***p*** |
| --- | --- | --- | --- | --- |
| **Environment (Scampia)** | 0.460 | 0.168 | 2.743 | .006 |
| **Sex (Male)** | 0.171 | 0.167 | 1.022 | .307 |
| **Age** | 0.526 | 0.059 | 8.924 | < .001 |
| Multiple *R^2^* = 0.210, Adjusted R-squared = 0.203 | | | | |

**Table 22.** Regression model for effect of environment on total scores for social STM.

| **Variable** | ***b*** | ***SE*** | ***t*** | ***p*** |
| --- | --- | --- | --- | --- |
| **Environment (Scampia)** | 6.897 | 1.275 | 5.412 | < .001 |
| **Sex (Male)** | −0.143 | 1.272 | −0.112 | .911 |
| **Age** | 5.159 | 0.449 | 11.490 | < .001 |
| Multiple *R^2^* = 0.327, Adjusted R-squared = 0.321 | | | | |

**Table 23.** Regression model for effect of environment on total scores for social WM.

| **Variable** | ***b*** | ***SE*** | ***t*** | ***p*** |
| --- | --- | --- | --- | --- |
| **Environment (Scampia)** | 3.754 | 1.373 | 2.734 | 0.007 |
| **Sex (Male)** | 1.462 | 1.369 | 1.067 | 0.287 |
| **Age** | 4.562 | 0.483 | 9.450 | < .001 |
| Multiple *R^2^* = 0.228, Adjusted R-squared = 0.221 | | | | |

**Table 24.** Regression model for effect of PSS on span scores for social STM.

| **Variable** | ***b*** | ***SE*** | ***t*** | ***p*** |
| --- | --- | --- | --- | --- |
| **Intercept** | 1.695 | 0.454 | 3.732 | < .001 |
| **PSS** | −0.011 | 0.016 | −0.661 | .509 |
| **Sex (Male)** | −0.108 | 0.144 | −0.750 | .453 |
| **Age** | 0.495 | 0.051 | 9.692 | < .001 |
| Multiple *R^2^* = 0.220, Adjusted R-squared = 0.213 | | | | |

**Table 25.** Regression model for effect of PSS on span scores for social WM.

| **Variable** | ***b*** | ***SE*** | ***t*** | ***p*** | |
| --- | --- | --- | --- | --- | --- |
| **Intercept** | 0.138 | 0.532 | 0.259 | .796 | |
| **PSS** | 0.010 | 0.019 | 0.522 | .602 | |
| **Sex (Male)** | 0.143 | 0.169 | 0.849 | .396 | |
| **Age** | 0.525 | 0.060 | 8.674 | < .001 | |
| Multiple *R^2^* = 0.193, Adjusted R-squared = 0.186 | | | | |  |

**Table 26.** Cohen's *d* effect size by test type.

| **Task** | **Scampia v. Pozzuoli** |
| --- | --- |
| **Verbal STM** | *d* = 0.041, *SE* = 0.109, 95% CI = [−0.178, 0.252] |
| **Verbal WM** | *d* = 0.165, *SE* = 0.112, 95% CI = [−0.055, 0.385] |
| **Visuo-spatial STM** | *d* = −0.011, *SE* = 0.110, 95% CI = [−0.231, 0.202] |
| **Visuo-spatial WM** | *d* = −0.148, *SE* = 0.109, 95% CI = [−0.362, 0.066] |
| **Social STM** | *d* = 0.597, *SE* = 0.111, 95% CI = [0.384, 0.819] |
| **Social WM** | *d* = 0.272, *SE* = 0.109, 95% CI = [0.057, 0.484] |


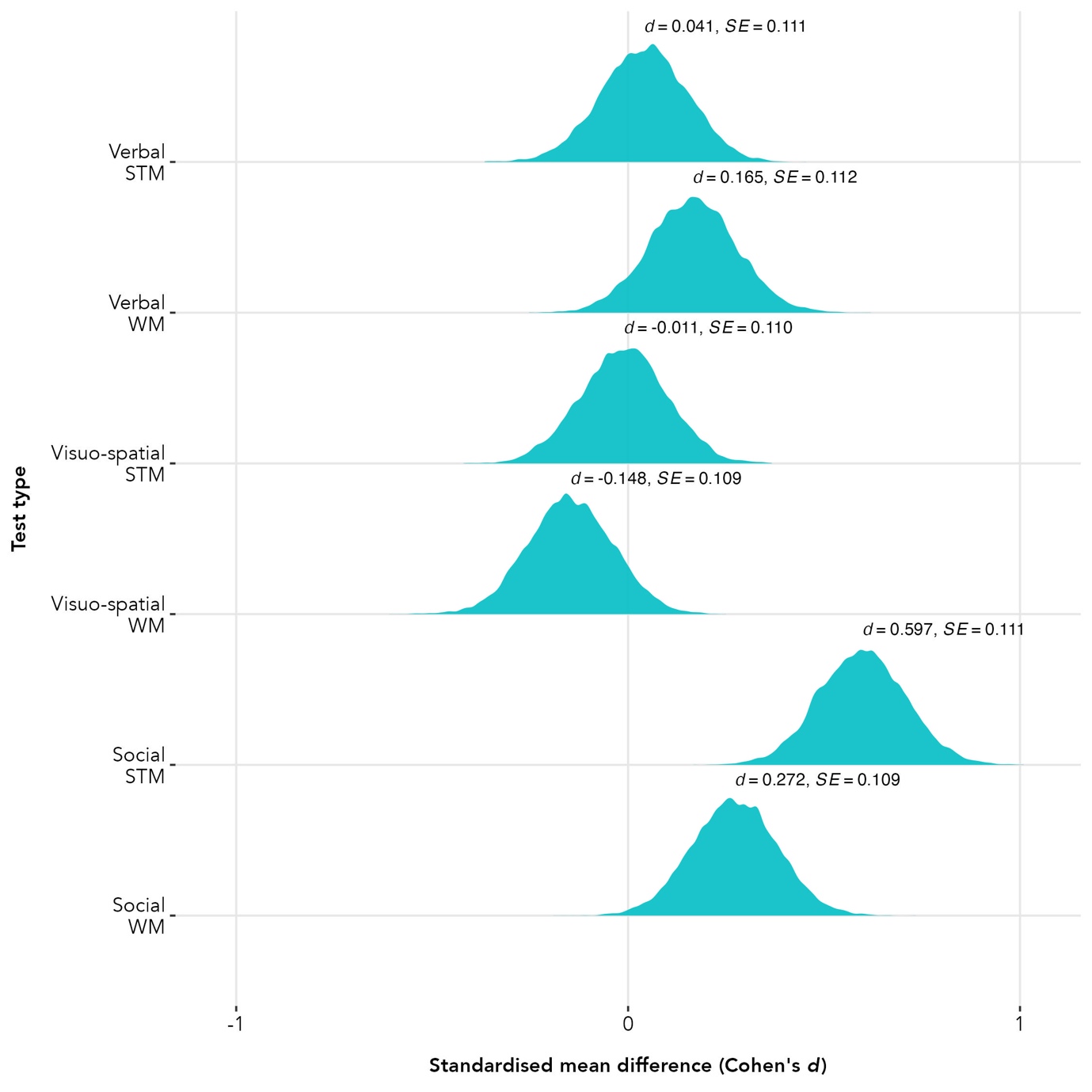


Figure 1. Effect sizes (Cohen’s d) for environment comparisons across test types; effect sizes indicate the “effect” of the Scampia environment as compared to the Pozzuoli environment.
